# Effects of a Red-Ginger-Based Multi-Nutrient Supplement on Optic Nerve Head Blood Flow in Open-Angle Glaucoma

**DOI:** 10.3390/nu18010140

**Published:** 2026-01-01

**Authors:** Akiko Hanyuda, Satoru Tsuda, Nana Takahashi, Naoki Takahashi, Kota Sato, Toru Nakazawa

**Affiliations:** 1 Department of Ophthalmology, Keio University School of Medicine, Shinanomachi, Shinjuku-ku 160-8582, Tokyo, Japan; akihanyu@keio.jp; 2 Department of Ophthalmology, Tohoku University Graduate School of Medicine, Aoba-ku, Sendai 980-8574, Miyagi, Japan; 3 Epidemiology and Prevention Group, Center for Public Health Sciences, National Cancer Center, Tsukiji, Chuo-ku 104-0045, Tokyo, Japan; 4 Division of Ophthalmic Precision Medicine Development, United Centers for Advanced Research and Translational Medicine (ART), Tohoku University Graduate School of Medicine, Aoba-ku, Sendai 980-8575, Miyagi, Japan; 5 Department of Advanced Ophthalmic Medicine, Tohoku University Graduate School of Medicine, Aoba-ku, Sendai 980-8574, Miyagi, Japan; 6 Department of Retinal Disease Control, Tohoku University Graduate School of Medicine, Aoba-ku, Sendai 980-8574, Miyagi, Japan

**Keywords:** aging, dietary supplement, glaucoma, ocular perfusion, red ginger

## Abstract

**Objectives**: Glaucoma is an age-related neurodegenerative disease, characterized by retinal ganglion cell loss and progressive visual field deterioration. Beyond intraocular pressure (IOP), vascular and metabolic dysregulation contributes to optic nerve head (ONH) ischemia and neuronal vulnerability. Nutritional factors with antioxidative and vasodilatory properties may help preserve ocular perfusion. This study investigated the acute and subacute effects of a single dose of a dietary supplement containing red ginger extract (*Zingiber officinale* var. *rubra*), lutein, and vitamin B6 on ONH blood flow in patients with open-angle glaucoma (OAG). **Methods**: A retrospective self-controlled study was conducted at Tohoku University Hospital between August 2023 and March 2025. ONH blood flow was quantified using a laser speckle flowgraphy (LSFG) baseline one hour after and one month after continuous oral supplementation in patients with OAG. Systemic parameters, ocular biometry, and concomitant glaucoma medications were recorded in medical charts. Relative mean blur rate (MBR) changes were analyzed using a linear mixed-effects model, accounting for repeated measures and inter-eye correlations. **Results**: Nineteen glaucoma patients (38 eyes) were included in the acute phase and 13 patients (26 eyes) completed the one-month follow-up. After adjusting for age and sex, a single oral dose of red ginger extract significantly increased the relative MBR at 1 h (106.9 ± 3.1%; *p* < 0.05), and this enhancement increased after 1 month of continuous intake (115.4 ± 6.7%; *p* < 0.05). Greater ONH perfusion was particularly prominent in eyes with shorter axial length. **Conclusions**: Oral supplementation was associated with acute and short-term increases in ONH blood flow in glaucomatous eyes. Although this study was a retrospective study without a placebo-controlled comparison group, our findings offer hypothesis-generating evidence that nutritional interventions may support ocular perfusion alongside conventional glaucoma management. Future prospective randomized controlled trials are required to confirm these associations.

## 1. Introduction

Glaucoma is a chronic, age-related neurodegenerative disease characterized by progressive retinal ganglion cell (RGC) loss and visual field deterioration, and remains a leading cause of irreversible blindness worldwide [1]. With the aging of global populations, its prevalence is projected to exceed 110 million by 2040, predominantly as primary open-angle glaucoma (POAG) [1]. Although intraocular pressure (IOP) reduction is the cornerstone of current management [2,3], disease onset and progression vary widely, even among patients with similar IOP levels [4]. This variability underscores the importance of aging-related vascular, metabolic, and oxidative mechanisms in glaucomatous neurodegeneration [5,6]. Proper optic nerve head (ONH) circulation is essential for maintaining axonal metabolism and RGC survival, as insufficient perfusion renders the lamina cribrosa region susceptible to ischemic injury and accelerates glaucomatous neurodegeneration [7]. Consequently, nutritional factors that are capable of improving microvascular function or reducing oxidative stress have gained increasing attention as potential adjunctive strategies for glaucoma management [8,9,10].

Among nutritional factors with vascular-modulating properties, red ginger (*Zingiber officinale* var. *rubra*) has attracted growing attention [11,12,13]. Its major bioactive constituents—6-gingerol, 8-gingerol, and 6-shogaol—exhibit antioxidative, anti-inflammatory, and vasodilatory effects through nitric oxide (NO)-mediated endothelial relaxation and suppression of endothelin-1 (ET-1)–induced vasoconstriction [11,12,13]. Notably, recent preclinical evidence demonstrated that red ginger extract improved retinal and ONH blood flow and attenuated ischemic injury in rats with ET-1-induced perfusion dysfunction [13]. This animal evidence provides a strong mechanistic basis for considering red ginger as a modulator of ocular microcirculation. Furthermore, red ginger offers rapid-onset vascular effects, making it suitable for evaluating acute ONH perfusion changes [13] compared with other nutrients (i.e., vitamins) that may accumulate in the retina for the relatively longer-term. However, clinical data investigating ginger or red ginger extracts in glaucoma patients remain extremely limited, and no prior studies have evaluated their acute effects on ONH perfusion.

In addition to red ginger, dietary carotenoids (lutein, zeaxanthin) [14], vitamins (B-complex) [15], and minerals [16] have been implicated in maintaining retinal metabolism and vascular homeostasis. Lutein accumulates in the retina and ONH, where it neutralizes reactive oxygen species, thereby contributing to neuroprotection [14]. Vitamin B-complex may reduce homocysteine levels, thereby mitigating the homocysteine-induced endothelial dysfunction implicated in impaired ocular perfusion [15]. These nutrients act synergistically to mitigate oxidative stress, improve endothelial function, and sustain ocular perfusion. However, the acute effects of functional food ingredients, such as red ginger extract, lutein, and vitamins, on ONH circulation have not been previously evaluated in human subjects with glaucoma.

Therefore, the present study aimed to investigate the effects of an oral dietary supplement containing extract from red ginger, lutein, and vitamin B6 on ONH blood flow in patients with open-angle glaucoma (OAG), using laser speckle flowgraphy (LSFG) [17,18] under clinical settings. By exploring the nutritional modulation of ocular perfusion, this study seeks to provide translational insights into diet-based adjunctive strategies for glaucoma management.

## 2. Materials and Methods

### 2.1. Study Design and Population

A retrospective self-controlled study was conducted in consecutive patients with confirmed OAG, who administered a single oral dose of a dietary supplement (*Clearvision^TM^ RC*, Rohto Pharmaceutical Co., Ltd., Osaka, Japan) that contained Flow-ginger^TM^ (extract derived from red ginger [*Zingiber officinale* var. *rubra*] 20 mg), lutein (20 mg), and vitamin B6 (7.2 mg), at Tohoku University Hospital (Miyagi, Japan) between August 2023 and March 2025. At the ophthalmology outpatient clinic, patients who administered oral dietary supplements underwent ophthalmologic evaluations, including ONH blood circulation before and after the intake of dietary supplements. Patients were excluded if they had any ocular disorders other than OAG or systemic diseases known to affect the visual field. The study protocol was approved by the Institutional Review Board of the Tohoku University Graduate School of Medicine (protocol code 2025-1-547). Because this study involved a retrospective design and posed minimal risk to participants, the Institutional Review Board granted a waiver of written informed consent, and all procedures adhered to the tenets of the Declaration of Helsinki.

### 2.2. Systemic and Ocular Examinations

Patients received a comprehensive ophthalmologic examination that included slit-lamp biomicroscopy, gonioscopic assessment, and dilated fundus examination, as well as IOP measurement and full static threshold perimetry.

IOP was assessed using Goldmann applanation tonometry (Carl Zeiss Meditec, Dublin, CA, USA), while patients remained on their existing topical glaucoma treatment. Axial length was measured with an optical biometer (OA-2000, TOMEY CORPORATION, Nagoya, Japan), and the circumpapillary retinal nerve fiber layer thickness (cpRNFLT) was measured with optical coherence tomography (DRI OCT Triton, Topcon, Inc., Tokyo, Japan).

Glaucomatous eyes were defined as those showing glaucomatous optic neuropathy with corresponding visual field defects, based on the Anderson–Patella criteria, as determined by glaucoma specialists. An abnormal visual field was defined as one meeting any of the following criteria: results outside normal limits on the glaucoma hemifield test; a cluster of three or more non-edge points on the pattern deviation plot depressed at the *p* < 0.05 level, with at least one point at the *p* < 0.01 level; or a corrected pattern standard deviation that was significantly abnormal at the *p* < 0.05 level. Standard automated perimetry was conducted using the Swedish interactive threshold algorithm (SITA) as a standard strategy of the 24-2 program on the Humphrey Field Analyzer (Carl Zeiss Meditec, Dublin, CA, USA). Glaucomatous eyes with an open anterior chamber angle (without exfoliation material) and IOP ≤ 21 mmHg and >21 mmHg were normal-tension glaucoma (NTG) and high-tension glaucoma (HTG) [3].

Pupillary dilation was achieved using 0.4% tropicamide. To enhance consistency and reproducibility, participants were asked to briefly move away from the instrument and reposition themselves after each image acquisition. During imaging, participants fixated on a target at the center of the visual field, and the examiner adjusted the fixation to align the eye’s position as needed. The imaging field was centered midway between the optic disk and the macula. The eye position was stored by the capture software (LSFG-NAVI, version 3.1.59.0; Softcare Ltd., Fukutsu, Japan) to ensure consistent imaging areas across subsequent sessions.

When the image focus was inadequate, dark vertical lines on the live image were used for manual adjustment. The mean blur rate (MBR) parameters of the optic disk were calculated automatically by the analysis software. The optic disk margin was delineated using a circular rubber band tool, and the position of each region of interest was saved and reapplied in follow-up measurements for the same patient. The change in ONH blood flow was defined as relative ratio of MBR (% MBR), which was calculated as (MBR after oral administration/MBR baseline) × 100%.

Systolic and diastolic blood pressures (SBP and DBP) were measured, after which ONH circulation was assessed three times, using LSFG. For statistical analysis, we used the mean values of these three measurements. Information regarding the use of topical antiglaucoma medications was also recorded.

### 2.3. Statistical Analyses

As this was an exploratory analysis, sample size was not predetermined. Data were reported as the mean and standard deviation (SD) for continuous variables and percentages for categorial variables. We used a linear mixed-effects model to compare the statistical difference in % MBR at each phase of the red ginger extract, to account for repeated measures and inter-eye correlations. To determine the differences in patient demographics and clinical presentations in MBR responses to the dietary supplement, we used Student’s *t*-test, chi-squared, and Fisher’s exact tests, as appropriate. Stratified analyses by axial length (the cut-off point of 25.9 mm was the mean value of the study participants) were additionally conducted. We also examined whether baseline characteristics were varied between the supplement-continuation and supplement-discontinuation groups. SAS (version 9.4, SAS Institute, Cary, NC, USA) was used for all statistical analyses. Two-tailed *p*-values < 0.05 indicated statistical significance.

## 3. Results

### 3.1. Patient Characteristics

In total, thirty-eight eyes from 19 patients (10 men and 9 women) with OAG were included. The type of glaucomatous eyes was NTG for 20, HTG for 11, and secondary glaucoma (steroid-induced glaucoma) for 7. The mean age was 60.2 ± 9.0 and IOP was 11.8 ± 3.7 mmHg. Patients were myopic (mean axial length of 25.9 ± 1.7 mm). The baseline SBP, DBP, and pulse rate were 122.8 ± 18.0, 70.3 ± 14.3, and 66.3 ± 9.2, respectively (Table 1).

### 3.2. ONH Blood Flow After the Supplement Intake

**Figure 1:**
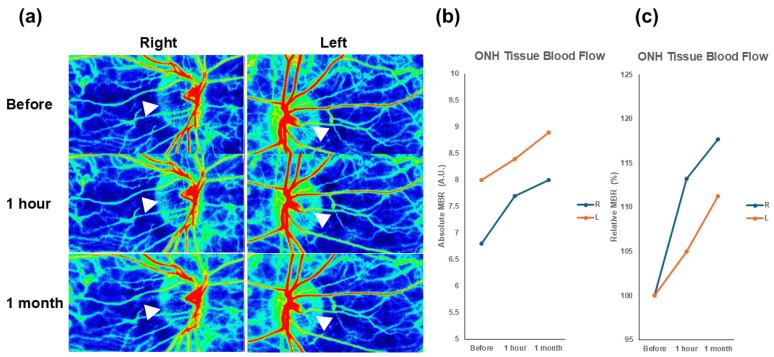
Changes in optic nerve head blood flow in a patient with normal-tension glaucoma, assessed using laser speckle flowgraphy. (**a**) Representative color map images obtained using LSFG at baseline, 1 h after supplementation and 1 month after continued daily intake. Warmer colors indicate a higher blood flow, with the ONH region being automatically segmented by the LSFG Analyzer software (version 3.1.59.0, Fukutsu, Japan). The white triangle symbol indicates the improvement in ONH blood flow following supplementation. (**b**) Absolute changes in MBR for both eyes. (**c**) Relative (%) changes in MBR for both eyes. Abbreviations: L, light eye; LSFG, laser speckle flowgraphy; MBR, mean blur rate; ONH, optic nerve head; R, right eye.

For a total of 38 eyes, the mean, ONH MBR, was 7.8 ± 2.9 AU at baseline, which significantly increased to 8.3 ± 3.1 AU at 1 h after the supplement intake. Figure 1a showed representative color map images of ONH blood flow after supplement intake and its changes in MBR (Figure 1b,c).

**Figure 2:**
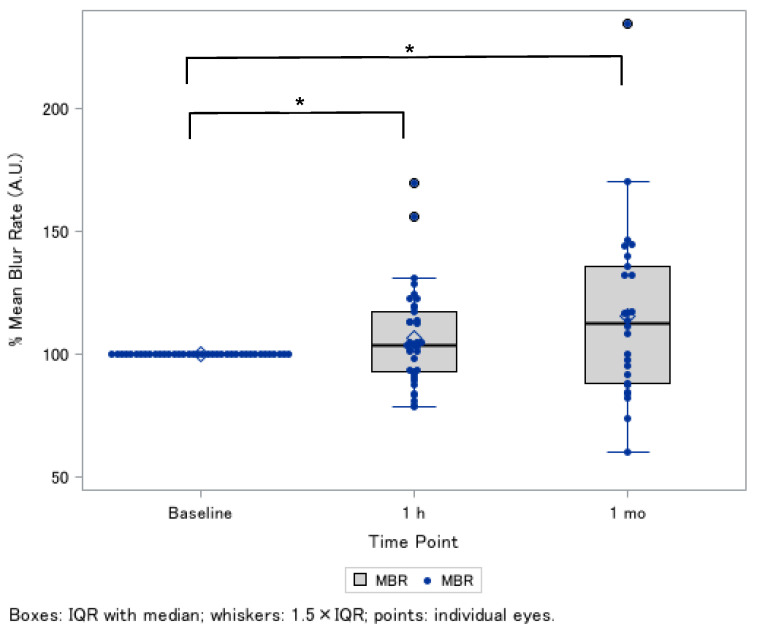
Changes in optic nerve head blood flow after oral supplement intake (post-1 h and 1 month) ^†^. Changes in optic nerve head blood flow showed relative MBR changes (%) at baseline, 1 h after supplementation and 1 month after continued intake. Each point represents an individual eye, and lines illustrate within-eye trajectories over time. ^†^ Thirty-eight eyes were included at baseline and at the 1 h time point, and only twenty-six eyes were followed up at the 1 month time point. * *p* < 0.05. Abbreviations: IQR, interquartile range; MBR, mean blur rate.

In the linear-mixed model, after being adjusted for several confounding factors (i.e., age, sex, axial length, mean arterial pressure, and IOP), relative MBR was 7.0 ± 3.0% increase at 1 h and 14.9 ± 6.7% at 1 month after continuous oral supplementation (Table 2 and Figure 2). Baseline characteristics were generally similar between the supplement-continued and -discontinued groups, except IOP was lower in the supplement-discontinued group (Supplementary Table S1). Also, relative MBR after supplement intake was likely to be greater in those with the supplement continued than those with the supplement discontinued (Supplementary Table S2).

We further examined differential factors associated with the responses of the ONH blood flow after supplementation (Table 3). Among various demographic and clinical features, there was a significant difference in the axial length, where greater ONH blood flow improvement was observed in those with a shorter axial length (*p* = 0.006). No other ocular and systemic factors differed between these groups (*p* > 0.05).

**Figure 3:**
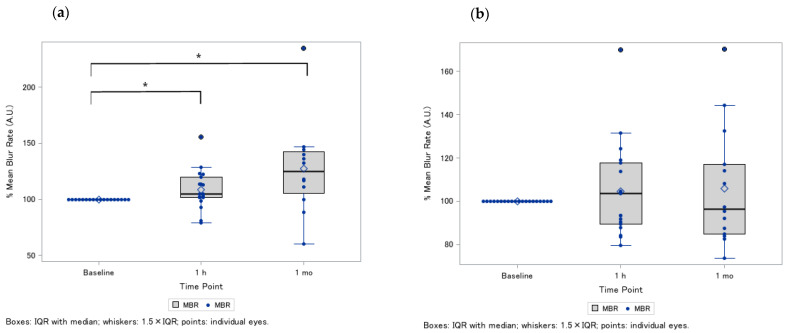
Changes in optic nerve head blood flow after oral supplement intake (post-1 h and 1 month), stratified by axial length ^†^. (**a**) Eyes with a shorter axial length, showing relative MBR changes (%) at baseline, 1 h after supplementation, and 1 month after continued intake. Each point represents an individual eye, and lines illustrate within-eye trajectories over time. (**b**) Eyes with a longer axial length, presented using the same plotting format, allowing for comparison of perfusion responses between axial-length strata. Relative MBR (%) was calculated as follows: (post-intake MBR/baseline MBR) × 100. ^†^ Thirty-eight eyes were included at baseline and at the 1 h time point, and only twenty-six eyes were followed up at the 1 month time point. The cut-off point of the axial length was the mean value of 25.9 mm. * *p* < 0.05. Abbreviations: IQR, interquartile range; MBR, mean blur rate.

In the exploratory analysis, we further conducted stratified analysis for the effects of ONH blood flow on dietary supplementation, according to the axial length. Greater ONH blood flow was observed in those with a shorter axial length (Figure 3a), but not in those with a longer axial length (Figure 3b).

## 4. Discussion

In this retrospective self-controlled study, we demonstrated that a single oral administration of a dietary supplement containing a red ginger extract was associated with increased ONH blood flow, as assessed by LSFG in patients with OAG. Furthermore, continued supplementation over one month maintained an elevated ONH blood flow, suggesting a sustained association in the short term. Although retrospective in nature, and we did not assess the observed perfusion changes to any single ingredient since the supplement contained multiple bioactive components (including red ginger extract, lutein, and vitamin B6), this study provides human evidence supporting the vascular efficacy of a red ginger-derived supplement in glaucomatous eyes.

Red ginger contains multiple phenolic compounds, including 6-gingerol, 8-gingerol, and 6-shogaol, that exhibit antioxidative, anti-inflammatory, and vasodilatory properties [11,12,13]. These compounds promote endothelial NO synthase activity, inhibit ET-1–mediated vasoconstriction, and reduce oxidative stress in vascular endothelium [11,12,13]. In a recent experimental study, Takahashi et al. demonstrated that red ginger extract restored retinal and ONH blood flow and ameliorated ET-1-induced ischemic injury in rats [13]. Our human data corroborate these experimental findings, suggesting that red ginger supplementation may enhance ocular perfusion through NO-dependent vasodilation and endothelial stabilization. Beyond vascular regulation, red ginger polyphenols and trace minerals may support the mitochondrial energy metabolism and protect RGCs from oxidative injury [15,16]. Given that mitochondrial dysfunction and microvascular dysregulation are central to glaucomatous neurodegeneration, these bioactive compounds may confer indirect neuroprotection via improved perfusion and reduced oxidative burden.

Although NO-dependent vasodilation is a plausible mechanism underlying the observed increase in ONH perfusion, the supplement used in this study (*Clearvision^TM^ RC*, Rohto Pharmaceutical Co., Ltd., Osaka, Japan) also contained lutein, vitamin B6, and minerals—all of which may modulate vascular function through overlapping or complementary pathways. Lutein is known to accumulate in the retina and ONH, where they neutralize reactive oxygen species and enhance microvascular reactivity [19]. Several clinical studies have reported that lutein supplementation improves ocular blood flow indices and retinal vascular responsiveness in healthy subjects [14]. B-vitamins—particularly B_6_, B_12_, and folate—may decrease homocysteine levels, mitigating endothelial injuries linked to impaired ocular perfusion, independent of NO pathways [20,21,22]. Thus, the perfusion changes observed in this study should not be attributed exclusively to red ginger or NO enhancement but rather should be considered the possible result of synergistic interactions among multiple antioxidative and vasculotropic nutrients. Although we did not evaluate the functional (i.e., visual field progression) or structural (i.e., macular pigment density) outcomes after supplementation, the collective evidence supports the concept that nutritional modulation of vascular and oxidative pathways may help stabilize ocular circulation and potentially slow age-related retinal neurodegeneration. Future clinical trials are warranted to confirm these effects and to determine long-term visual benefits.

The acute rise in ONH blood flow following a single oral dose of supplement highlights the dynamic capacity of ocular microvasculature to respond to dietary bioactives. This observation is particularly relevant for NTG (the major type of glaucoma included in this study), in which vascular dysregulation and oxidative stress play dominant roles despite normal IOP [23,24]. Flammer and his colleagues described a subset of patients with NTG who present with systemic vascular dysregulation, characterized by cold extremities, migraines, and increased peripheral vasoconstrictive responses [25]. Improving ONH perfusion by reversing these excessive vasoconstrictive tendencies therefore represents a promising therapeutic target for glaucoma management. Indeed, our previous clinical studies demonstrated that the intake of *Kampo* medicines—traditional Japanese botanical formulations—significantly increased ONH blood flow in both healthy individuals [26] and patients with Flammer syndrome [27]. Given this pathophysiological context, supplementation with bioactive compounds that exert vasodilatory and endothelial-stabilizing effects—such as red ginger extract—may hold therapeutic potential for glaucomatous patients, although further clinical studies are warranted.

Although the present study demonstrated a 6.9% acute increase and a 15.4% one-month increase in relative MBR, the clinical relevance of these changes must be interpreted carefully. Prior LSFG work by Kiyota et al. showed that even modest reductions in the ONH blood flow predicted subsequent RNFL thinning in OAG [17], suggesting that relatively small perfusion differences may contribute to clinically meaningful progression. In this context, the magnitude of improvement observed in our study could reflect an enhanced microvascular reserve that may, in theory, translate into reduced vulnerability to ischemic stress. Nonetheless, given that disease progression typically occurs over years and MBR reflects microvascular dynamics, rather than direct neuroprotective outcomes, the one-month follow-up does not allow for assessment of structural preservation or functional improvement. Future randomized, double-blind, placebo-controlled trials with extended follow-up (at least six–twelve months), serial OCT and LSFG assessments, and functional endpoints are essential to evaluate whether nutritional modulation of ONH blood flow can meaningfully alter the trajectory of glaucomatous damage.

Interestingly, in the present study, the improvement in ONH blood flow following supplementation was more pronounced in eyes with a shorter axial length, whereas eyes with a longer axial length exhibited lower or no perfusion change. This axial length-dependent response suggests that ocular structural remodeling in myopia may influence vascular reactivity. Myopic eyes are known to have thinning of the lamina cribrosa, stretching of peripapillary scleral tissue, and increased vascular resistance due to elongation of the posterior pole, all of which can impair microvascular autoregulation [28,29,30,31].

Aizawa et al. demonstrated a close correlation between ONH microcirculation and both structural and functional parameters in myopic glaucoma, indicating that reduced baseline perfusion is closely linked to mechanical deformation and vascular compromise in elongated eyes [32]. Similarly, Huang et al. recently reported that increasing axial length—more than chronological aging—was independently associated with decreased RNFL thickness and superficial vessel density in non-glaucomatous eyes [33]. These observations support the concept that axial elongation itself attenuates vascular adaptability, possibly through alterations in vessel wall elasticity, choroidal thinning, or impaired endothelial responsiveness.

Therefore, the greater ONH blood-flow enhancement observed in eyes with a shorter axial length may reflect the preserved vascular reactivity in less structurally stretched ocular tissues, whereas the blunted response in highly myopic eyes may indicate limited hemodynamic reserve, due to chronic mechanical and vascular remodeling. This finding highlights the importance of considering the axial length as a modifying factor in ocular perfusion studies and suggests that early vascular intervention or nutritional modulation may be particularly beneficial before irreversible myopic microvascular remodeling occurs.

Aging is accompanied by endothelial dysfunction, reduced NO bioavailability, and mitochondrial decline, all of which impair the autoregulation of ocular blood flow [34,35]. These vascular changes parallel those observed in systemic neurodegenerative diseases such as Alzheimer’s and Parkinson’s disease, which also share pathways of oxidative stress and vascular dysregulation [36,37]. Nutritional interventions that enhance endothelial health—such as polyphenol-rich foods, omega-3 fatty acids, and antioxidant micronutrients—have been proposed as low-risk adjunctive strategies to preserve neurovascular integrity [38].

The present study has several limitations. First, its retrospective, self-controlled design, modest sample size, and absence of a placebo-controlled arm substantially limit the ability to establish causal relationships. As a result, the observed associations between supplementation and short-term perfusion changes should be interpreted as exploratory. Second, the duration and sustainability of the observed perfusion improvements beyond one month remain uncertain. In a post hoc power calculation, based on the observed variance and effect sizes in the relative MBR in this study, the available sample provided approximately 53% power to detect the 1 h increase and 61% power to detect the 1 month increase at a two-sided α = 0.05. Although the bootstrap analysis, with 1000 resamples at subject levels, confirmed the reliability of the observed increase in ONH perfusion (the mean increase in relative MBR at 1 h was 106.4% [95% bootstrap CI: 79.5–155.8] and at 1 month was 115.2% [95% bootstrap CI: 73.7–170.2], this study was underpowered for smaller effects and should be regarded as an exploratory pilot investigation. In addition, there might be a selection bias, since those with the supplement being continued were likely to have a greater MBR response after supplement intake. Third, because the supplement contained multiple active components (e.g., red ginger extract 20mg, lutein 20mg, and vitamin B_6_ 7.2 mg), the specific contribution of each nutrient cannot be independently determined—although mechanistically, red ginger is expected to play the primary role in enhancing ocular perfusion [13]. Future randomized controlled trials with larger cohorts are warranted to confirm these findings, assess the dose–response and long-term effects, and examine correlations with functional (e.g., visual field progression, pattern electrophysiology parameters) and structural (e.g., retinal oxygen saturation, choroidal thickness) outcomes. Additionally, integrating systemic vascular biomarkers and oxidative stress indices may help to elucidate the molecular mechanisms linking nutritional modulation and ocular microcirculation.

## 5. Conclusions

In conclusion, dietary supplementation, including red ginger extract, lutein, and vitamin B6 acutely and chronically, was associated with increased ONH blood flow in patients with OAG. While these findings partly align with preclinical evidence demonstrating red ginger-mediated vasodilation and neuroprotection effects [13], this study was limited to infer the causal association. Instead, the results generate preliminary hypotheses, suggesting that nutritional bioactives may have a complementary role in supporting ocular perfusion in glaucoma. Further controlled trials are warranted to confirm these effects and to integrate nutritional vascular modulation into comprehensive glaucoma management.

## Figures and Tables

**Table 1:** Baseline demographic and clinical characteristics of the study participants (*n* = 19) and their glaucomatous eyes (*n* = 38).

**Demographic Features of Included Subjects (Total 19 Patients)**	
Age, mean (SD), year	60.2 (9.0)
Male, *n* (%)	10 (52.6)
Systolic blood pressure, mean (SD), mmHg	122.8 (18.0)
Diastolic blood pressure, mean (SD), mmHg	70.3 (14.3)
Pulse rate, mean (SD), bpm	66.3 (9.2)
Number of glaucoma eye drops, mean (SD)	4.3 (0.9)
**Clinical features of eyes with glaucoma (total 38 eyes)**	
Types of glaucoma	
High-tension glaucoma (≥21 mm Hg), *n* (%)	11 (28.9)
Normal-tension glaucoma (<21 mmHg), *n* (%)	20 (52.6)
Steroid-induced glaucoma, *n* (%)	7 (18.4)
Intraocular pressure, mean (SD), mmHg	11.8 (3.7)
Axial length, mean (SD), mm	25.9 (1.7)
Mean deviation, mean (SD), dB	−14.0 (8.7)
Circumpapillary retinal nerve fiber layer thickness, mean (SD), µm	66.4 (16.4)
Ganglion cell complex layer, mean (SD), µm	75.1 (12.9)
Ocular perfusion pressure, mean (SD), mmHg	46.7 (9.9)
Mean blur rate, mean (SD), AU	7.8 (2.9)

Abbreviations: SD, standard deviation.

**Table 2:** Changes in optic nerve head blood flow after oral supplement intake (post-1 h and 1 month) ^†^.

Relative MBR ^¶^, % (Tissue-Area MBR, AU)	Post-1 h (*n* = 38 Eyes)	Post-1 Month (*n* = 26 Eyes)
Age, sex-adjusted (Model 1)	106.9 ± 3.1 *	115.4 ± 6.7 *
Model 1 + axial length-adjusted	106.9 ± 3.1 *	115.4 ± 6.7 *
Model 1 + axial length, MAP, IOP-adjusted	107.0 ± 3.0 *	114.9 ± 6.7 *

^†^ Thirty-eight eyes were included at baseline and at the 1 h time point, and only twenty-six eyes were followed up at the 1 month point. ^¶^ Relative MBR was the percentage of baseline MBR. * *p* < 0.05. Abbreviations: IOP, intraocular pressure; MAP, mean arterial pressure and MBR, mean blur rate.

**Table 3:** Factors associated with changes in optic nerve head blood flow after oral supplement intake at 1 h time point.

Demographic Features of Included Subjects (Total 19 Patients)	ONH Blood Flow (Up)	ONH Blood Flow (Down)	*p*-Value *
Age, mean (SD), year	61.5 (8.7)	57.8 (9.3)	0.24
Male, *n* (%)	6 (15.8)	4 (10.5)	0.50
Systolic blood pressure, mean (SD), mmHg	123.1 (18.3)	123.2 (18.7)	0.99
Diastolic blood pressure, mean (SD), mmHg	69.5 (16.0)	71.9 (11.6)	0.63
Pulse rate, mean (SD), bpm	65.8 (10.1)	67.5 (7.9)	0.62
Number of glaucoma eye drops, mean (SD)	4.1 (0.9)	3.8 (0.8)	0.39
**Clinical features of eyes with glaucoma (total 38 eyes)**			
Types of glaucoma			0.85
High-tension glaucoma (≥21 mm Hg), *n* (%)	14 (36.8)	6 (15.8)	
Normal-tension glaucoma (<21 mmHg), *n* (%)	7 (18.4)	4 (10.5)	
Steroid-induced glaucoma, *n* (%)	4 (10.5)	3 (7.8)	
Intraocular pressure, mean (SD), mmHg	12.3 (0.7)	11.2 (1.1)	0.36
Axial length, mean (SD), mm	25.4 (1.4)	27.0 (1.8)	**0.006**
Mean deviation, mean (SD), dB	−13.6 (8.7)	−13.4 (8.2)	0.96
Circumpapillary retinal nerve fiber layer thickness, mean (SD), µm	65.6 (14.8)	69.5 (19.0)	0.49
Ganglion cell complex layer, mean (SD), µm	75.0 (12.5)	76.7 (13.6)	0.72
Ocular perfusion pressure, mean (SD), mmHg	45.9 (10.3)	48.2 (9.7)	0.52

* *p* < 0.05 was presented in bold. Abbreviations: ONH, optic nerve head; SD, standard deviation.

## Data Availability

The data presented in this study are available on request from the corresponding author. The data are not publicly available due to ethical restrictions.

## Supplementary Materials

The following supporting information can be downloaded at: https://www.mdpi.com/article/10.3390/nu18010140/s1. Table S1: Comparison of baseline demographic and clinical characteristics of the study participants according to supplement continuation status; Table S2: Changes in optic nerve head blood flow after oral supplement intake (post-1 h).

## Abbreviations

The following abbreviations are used in this manuscript:

cpRNFLT	Circumpapillary retinal nerve fiber layer thickness
DBP	Diastolic blood pressure
ET-1	Endothelin-1
HTG	High-tension glaucoma
IOP	Intraocular pressure
LSFG	Laser speckle flowgraphy
MBR	Mean blur rate
NO	Nitric oxide
NTG	Normal-tension glaucoma
OAG	Open-angle glaucoma
OCT	Optical coherence tomography
ONH	Optic nerve head
POAG	Primary open-angle glaucoma
RGC	Retinal ganglion cell
SBP	Systolic blood pressure
SD	Standard deviation
SITA	Swedish Interactive Threshold Algorithm
