# Supplementary material for: Effects of a Red-Ginger-Based Multi-Nutrient Supplement on Optic Nerve Head Blood Flow in Open-Angle Glaucoma
Source: Nutrients. 2026 Jan 1;18(1):140. doi: 10.3390/nu18010140 (PMC12787861; doi:10.3390/nu18010140)
Supplement: Supplementary file 1 [file nutrients-18-00140-s001.zip › nutrients-3990681-supplementary.pdf]

Supplementary Materials

Title: Effects of a Red-Ginger-Based Multi-Nutrient Supplement on Optic Nerve Head Blood Flow in Open-Angle Glaucoma

Correspondence to: Toru Nakazawa, MD, PhD

Department of Ophthalmology, Tohoku University Graduate School of Medicine, Seiryo-machi, Aoba-ku,
Sendai, Miyagi 980-8574, Japan

Supplementary Table S1. Comparison of baseline demographic and clinical characteristics of the study participants according to supplement continuation status

Demographic features of included subjects (total 19 patients)	Supplement Discontinued (n=12 eyes)	Supplement Continued (n=26 eyes)	P-value
Age, mean (SD), year	59.7 (8.0)	60.5 (9.4)	0.80
Male, n (%)	4 (66.7)	6 (46.2)	0.41
Systolic blood pressure, mean (SD), mmHg	127.8 (20.6)	120.5 (16.6)	0.25
Diastolic blood pressure, mean (SD), mmHg	75.0 (12.4)	68.2 (14.8)	0.17
Pulse rate, mean (SD), bpm	70.0 (8.6)	64.6 (9.1)	0.09
Number of glaucoma eye drops, mean (SD)	3.8 (0.7)	4.1 (1.0)	0.46
**Clinical features of eyes with glaucoma (total 38 eyes)**			
Types of glaucoma			0.70
High-tension glaucoma (≥21mm Hg), n (%)	2 (16.7)	9 (34.6)	
Normal-tension glaucoma (<21mmHg), n (%)	8 (66.7)	12 (46.2)	
Steroid-induced glaucoma, n (%)	2 (16.7)	5 (19.2)	
Intraocular pressure, mean (SD), mmHg	10.0 (2.8)	12.6 (3.9)	0.04
Axial length, mean (SD), mm	26.3 (2.1)	25.7 (1.5)	0.36
Mean deviation, mean (SD), dB	-17.0 (9.0)	-12.6 (8.4)	0.15
Circumpapillary retinal nerve fiber layer thickness, mean (SD), μm	59.3 (16.3)	69.7 (15.7)	0.07
Ganglion cell complex layer, mean (SD), μm	72.6 (14.4)	76.1 (12.4)	0.47
Mean blur rate, mean (SD), AU	7.6 (2.7)	7.9 (3.0)	0.76

Abbreviations: SD, standard deviation.

Supplementary Table S2. Changes in optic nerve head blood flow after oral supplement intake (post-1 hour)†

Relative MBR¶, % (Tissue-area MBR, AU)	Supplement Discontinued (n=12 eyes)	Supplement Continued (n=26 eyes)
Age, sex-adjusted (Model 1)	98.2±7.0	110.9±3.3
Model 1 + Axial length-adjusted	96.6±6.1	110.8±3.3
Model 1 + Axial length, MAP, IOP-adjusted	96.5±6.0	110.9±3.4

†Thirty-eight eyes were included at baseline and at 1 hour time point and only twenty-six eyes were followed at 1 month point.

¶Relative MBR was the percentage of baseline MBR

Abbreviations: IOP, intraocular pressure; MAP, mean arterial pressure; MBR, mean blur rate.
